# Preserved cortical thickness, surface area and volume in adolescents with PTSD after childhood sexual abuse

**DOI:** 10.1038/s41598-020-60256-3

**Published:** 2020-02-24

**Authors:** Mirjam A. Rinne-Albers, Charlotte P. Boateng, Steven J. van der Werff, Francien Lamers-Winkelman, Serge A. Rombouts, Robert R. Vermeiren, Nic J. van der Wee

**Affiliations:** 1 Curium-LUMC, Academic Center for Child and Adolescent Psychiatry, Oegstgeest, the Netherlands; 2 Department of Psychiatry, Leiden University Medical Center (LUMC), Leiden, the Netherlands; 3 Leiden Institute for Brain and Cognition (LIBC), Leiden, the Netherlands; 4 Institute of Psychology and Education, Vrije Universiteit, Amsterdam, the Netherlands; 5 Department of Radiology, Leiden University Medical Center (LUMC), Leiden, the Netherlands; 6 Institute of Psychology, Leiden University, Leiden, the Netherlands

**Keywords:** Health care, Medical research

## Abstract

Exposure to childhood adverse events is associated with severe consequences for general health and structural and functional changes in the brain of its survivors. In order to unravel and in the end influence the pathway linking adversity and pathology, neuroimaging research is crucial. Up till now studies in minors are scarce and differ in type of adversity or methodology. Almost all studies report lower cortical thickness, but in a broad variety of regions. In this study we investigated cortical thickness measures and clinical data in a well circumscribed group of adolescents with PTSD related to childhood sexual abuse (CSA) (N = 21) and a healthy non-traumatised control group (N = 21). The ventromedial PFC (vmPFC), ACC, insula, and middle/superior temporal gyrus were chosen as ROI’s due to their respective roles in emotion and information processing. No significant effect of group was found for cortical thickness, surface area or volume in any of the ROIs. This is in line with the results of research in adult women with sexual abuse related PTSD, suggesting that this may be specific to this group, independent of age. Recent research points to differential biological and pathological consequences of different types of childhood adversity.

## Introduction

The experience of traumatic events in childhood is a strong predictor of psychiatric and somatic pathology later in life^1–3^, with substantial consequences for general health^4,5^. Recent epigenetic research further demonstrated the substantial impact of stress and trauma on the next generation^6^. In order to counter the negative effects of childhood psychological trauma, it is necessary to understand the neurobiological processes related to these trajectories. Results from preclinical and human studies link stress hormones like cortisol to structural changes in the brain, reflecting underlying alterations in neuron density and microstructure^7–9^. Psychological processes like threat processing, heightened emotional reactivity and altered emotional learning are thought to mediate between trauma exposure, structural changes in the brain and later psychopathology^10^. Understanding the developing and still malleable child or adolescent brain after trauma could provide an approach for thwarting negative consequences, particularly because even the adult brain is considered to be susceptible for structural changes after therapeutic interventions^11,12^. Structural neuroimaging research in traumatised children and adolescents may help to elucidate the effects of childhood adverse events on brain development, however, up till this moment such studies are scarce^13^.

Meta-analyses of structural MRI studies in adults with PTSD have reported smaller gray matter volume (GMV) in emotion and threat processing structures like the hippocampus, the anterior cingulate cortex (ACC) and the medial prefrontal cortex (mPFC)^14,15^. Apart from a smaller corpus callosum (CC), results of structural MRI studies in traumatised C&A however, are mixed and inconclusive^13^. Results from neuroimaging studies in adult victims of childhood trauma thus differ from findings in traumatised minors. While studies in adults with a history of childhood abuse or neglect consistently report smaller volume of the hippocampus compared to non-traumatised controls, this has not been replicated in minors^16,17^. In contrast, the decrease in volume of the corpus callosum, has consistently been reported in children and adolescents, but not in adults who have experienced childhood trauma^18,19^. Clearly, more research is needed in order to understand the neurodevelopmental trajectories related to trauma leading to the negative consequences in adulthood.

This study is part of the Emotional Pathways’ Imaging Study in Clinical Adolescents (EPISCA) project. Earlier research with the same groups, using VBM (Voxel Based Morphometry), DTI (Diffusion Tensor Imaging) and functional MRI techniques, have been published^19–26^ Cortical thickness is a relatively new neuroimaging analysis technique, which is considered complementary to VBM in studying grey mater integrity^27^.

Smaller cortical thickness in adult traumatised populations, mostly PTSD patients, has been reported in various brain regions like the mPFC, the ACC, the insula, and the superior and middle temporal gyrus^28–31^. Some studies even found an inverse correlation between cortical thickness and symptom severity^28,32^. A study in adult women with sexual abuse related PTSD, similar to our adolescent group, showed normal cortical thickness compared to healthy controls^33^. Greater cortical thickness has been associated with resilience in adults^34,35^. For those reasons, it is relevant to study cortical thickness in traumatised children and adolescents as well.

Cortical thickness studies in traumatised minors are, however, limited and show little concordance. To our knowledge there are only ten studies on cortical thickness in traumatised minors, almost all adolescents. In contrast to adult trauma research only two of these ten studies report about PTSD patients^36,37^, the others included minors having experienced different types of adversity. Studies found smaller cortical thickness, but not consistently, in several regions including the mPFC^38–42^, ACC^36,43,44^, insula^36,37,45^ temporal regions^38,39,41^, other frontal regions^39,42–45^ parahippocampal regions^39^ and parietal regions^41,42^ Exceptions are reported by Whittle *et al*.^42^ who compared maltreated with non-maltreated adolescents and found an association of maltreatment with accelerated as well as less thinning in frontal and precentral regions, and by Ahmed *et al*.^36^ who compared traumatised adolescents with and without PTSD and reported no significant difference in cortical thickness between groups in the insula, ACC, amygdala, CC and hippocampus using Freesurfer technique and a reduction in insula thickness when using Qdec. No clear distinction can be made between results from studies with inclusion based on the presence of PTSD or the presence of different types of trauma. Results of studies may vary due to differences in age and type of adversity.

In order to further elucidate the impact of trauma on the developing brain, we decided to examine cortical thickness, surface area and volume in our EPISCA sample, a well-defined group of adolescents with posttraumatic stress disorder based on childhood sexual abuse (CSA) with measures in non-traumatised controls. Based on the literature, we hypothesized differences in the following regions of interest (ROIs): the ventromedial PFC (vmPFC), ACC, insula, and middle/superior temporal gyrus, regions that are considered to play a role in emotion and information processing.

## Methods

### Participants

Participants were selected from the Emotional Pathways’ Imaging Study in Clinical Adolescents (EPISCA). EPISCA is a longitudinal MRI study in which adolescents with clinical depression, adolescents with a history of sexual trauma and healthy controls were followed over a six-month period in which they received treatment. The adolescents were assessed and underwent scanning at three time points: upon inclusion at baseline, three months after baseline, and six months after baseline^20,46^. The current study reports on cross-sectional baseline data from the adolescents with a history of sexual trauma and healthy non-traumatised controls. See also previous reports from neuroimaging research with the same population^19–26^. Inclusion criteria for the adolescents with a history of sexual trauma were: having experienced sexual abuse during their lifetime more than once by one or more perpetrators inside or outside the family, and being referred for treatment to the Psychotrauma Center of mental health institute GGZ Rivierduinen in Leiden or the child and adolescent psychotrauma center KJTC in Haarlem, both located in the Netherlands. Experienced psychotherapists in these specialised psychotrauma centers obtained the trauma histories from the adolescents as well as from their caregivers during clinical interviews. To objectify any abuse or neglect as well as risk for functional impairment and morbidity, we verified police reports, involvement of child welfare, and family custody or other child protection measures as to have an estimate of the severity and impact of problems. (For more details about the participants in the CSA- group see van Hoof *et al*.^46^).

Presence of PTSD was not an inclusion criterion, although clinical assessments (see below) showed that all patients but one were diagnosed with PTSD related to CSA. Inclusion criteria for the control group were: no current or past DSM-IV classifications, no clinical scores on validated mood and behavioural questionnaires, no history of traumatic experiences, and no current psychotherapeutic and/or psychopharmacological intervention of any kind. Exclusion criteria for all participants were: primary DSM-IV clinical diagnosis of attention-deficit hyperactivity disorder (ADHD), oppositional defiant disorder (ODD), conduct disorder (CD), pervasive developmental disorders, Tourette’s syndrome, obsessive–compulsive disorder, bipolar disorder, and psychotic disorders; current use of psychotropic medication other than stable use of SSRI’s, or amphetamine medication, but not on the day of scanning; current substance abuse; history of neurological disorders or severe head injury; age <12 or >21 years; pregnancy; left-handedness; IQ score <80 as measured by the Wechsler Intelligence Scale for Children (WISC)^47^ or adults^48^; and general MRI contraindications.

Fifty-four participants were included in the study: 32 healthy non-traumatised controls and 22 patients with a history of sexual trauma. From this group, two controls were excluded because of technical problems during scanning or poor imaging data quality, one participant with CSA because of complete segmentation failure. One control was excluded due to high scores on clinical symptom rating scales, and one control was excluded due to a history of sexual trauma that was not reported until the day of scanning. This resulted in a final sample of 21 adolescents with CSA and 28 controls.

Of the 21 CSA-participants, 20 fulfilled all PTSD criteria on the ADIS, while one had sufficient PTSD symptoms, but with limited interference. Since earlier research showed that persons with sub threshold PTSD in many aspects resemble PTSD patients, we decided to include this patient in the (CSA-related) PTSD group^49^.

The study was approved by the Medical Ethics Committees of the Leiden University Medical Center and written informed assent and consent was obtained from the participants and their parents respectively.

### Clinical assessment

A standardized set of instruments was used to assess symptomatology in both groups of adolescents.

The Anxiety Disorders Interview Schedule Child and Parent Versions (ADIS-C/P)^50^ are semi structured interviews for the classification of DSM-IV anxiety and depressive disorders in children. Classification is reached by a minimal interference score of 4 obtained by trained examiners based on the ADIS-C and ADIS-P. The ADIS is known to have good reliability and validity^51^ with reported strong test–retest reliability statistics for the ADIS-C/P for combined diagnoses (0.80–0.92) and individual diagnoses (0.62–0.88). The Trauma Symptom Checklist for Children (TSCC)^52^ is a 54-item self-report for children and adolescents aged 8 through 18 but often used up to 21 years^53,54^ which measures trauma-related symptoms. On a 4-point scale (never to almost all of the time), the adolescent indicates how often a thought, a feeling or a behaviour occurs. The items are grouped into six clinical scales. The clinical scales are Anxiety (Anx), Depression (Dep), Post-traumatic Stress (Pts), Sexual Concerns (Sc), Dissociation (Dis) and Anger (Ang). The TSCC total score is used as the main measure on post-traumatic symptomatology. Cronbach’s alpha coefficients reported range from 0.77 to 0.89 for subscales and 0.84 for the total scale. The questionnaire has extensively been studied, which has confirmed its good psychometric qualities^55,56^. The internal consistency of the TSCC subscales varied between 0.85 and 0.94, except for the Sexual Concerns subscale that measured 0.68.

The Adolescent Dissociative Experiences Scale (A-DES) contains 30 items to assess adolescents of 11–18 years of age for pathological dissociation. The A-DES items inquire about four domains reflecting basic aspects of dissociation: experiences of dissociative amnesia, depersonalization/derealisation, absorption/imaginative involvement and passive influence. The items are rated by the adolescent on an 11-point Likert-scale ranging from 0 = “never” to 10 = “always” with no midpoint scores. The total A-DES score is based on the mean of all item scores. A mean score of 4 or above on the A-DES signifies pathological dissociation^57^. The scale has good internal reliability and validity.

As brain development is known to be influenced by sexual development, corporal sexual development was measured with the self-report Puberty Development Scale^58^. The PDS consists of 5 items that are measured on a 5-point scale by the examiner: 1= pre-pubertal, 2= early pubertal, 3= mid-pubertal, 4= late pubertal, 5= post-pubertal. The PDS is considered a valuable instrument determining pubertal stage^59,60^.

Six subscales from the Wechsler Intelligence scales scores (picture completion, similarities, picture concepts, arithmetic, block design and comprehension) were converted into Full IQ estimates^20,46^.

All methods were performed in accordance with the relevant guidelines and regulations.

### MRI data acquisition

Images were acquired on a Philips 3T magnetic resonance imaging system (Philips Healthcare, Best, The Netherlands), equipped with a SENSE-8 head coil. Scanning took place at the Leiden University Medical Center. Prior to scanning, all participants were introduced to the scanning situation by lying in a dummy scanner and hearing scanner sounds. For each subject, a sagittal 3-dimensional gradient-echo T1-weighted image was acquired (repetition time = 9.8 ms; echo time = 4.6 ms; flip angle = 8°; 140 sagittal slices; no slice gap; field of view =256 × 256 mm; 1.17 × 1.17 × 1.2 mm voxels; duration =4:56 min) as part of a larger, fixed imaging protocol.

### Image processing and analysis

Cortical parcellations of 68 cortical grey matter regions, 34 regions in each cerebral hemisphere, were performed using FreeSurfer (Software version 5.3.0) based on the Desikan-Killiany atlas. In addition, two whole-hemisphere measures were extracted using FreeSurfer. The segmentations of all 68 cortical grey matter regions and the two whole-hemisphere measures were followed by a statistical outlier assessment and a visual inspection for artefacts and abnormal clinical findings, which was done independently by three different researchers using standardized ENIGMA-protocol: http://enigma.ini.usc.edu/protocols/imaging-protocols/protocol-for-quality-control-and-summary-statistics/ After visual inspection, both the left and right insula were excluded from further analyses due to frequent inadequate or complete failure of segmentation.

### Statistical analyses

Statistical analyses for all data were performed with the Statistical Package for Social Sciences Software (SPSS, version 25). Demographic and clinical characteristics were analysed using independent-sample t-tests. If the data did not meet the assumptions necessary for parametric analyses, which was the case for the total scores on the A-DES and TSCC, the non-parametric Mann-Whitney U-test was used. Categorical variables were assessed with the Chi-square test. All tests were performed with the significance set at p < 0.05.

**Figure 1:**
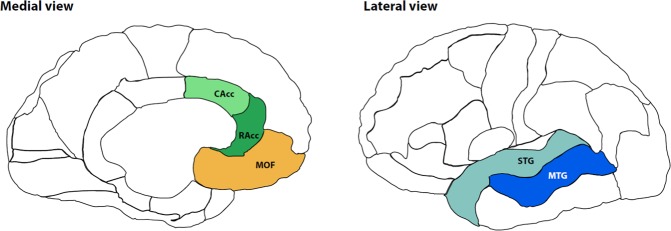
Regions of Interest (ROIs): medial orbitofrontal (MOF), caudal anterior cingulate cortex (CAcc), rostral anterior cingulate cortex (RAcc), superior temporal gyrus (STG), middle temporal gyrus (MTG). The left and right insula were excluded from further analyses due to frequent inadequate or complete failure of segmentation.

Total volume for each ROI was calculated by multiplying cortical thickness with surface area. The effect of PTSD on cortical morphometric measures in all ROIs was tested using a multivariate analyses of covariance (MANCOVA). Considering that age and intracranial volume are strongly correlated with cortical measures, both were included as covariates^61^. In line with previous research, puberty development (PDS score) was also accounted for in the model^19^. Three separate MANCOVAs were used to assess group differences, one for cortical thickness, one for surface area and one for volume of the selected ROI as dependent factors. Therefore, we corrected for multiple testing by dividing the p-value of 0.05 by 3 (p < 0.017). The ROIs included left and right ACC (caudal and rostral ACC in Freesurfer), middle temporal gyrus, superior temporal gyrus, vmPFC (medial orbitofrontal cortex in FreeSurfer). (Fig. 1). Post-hoc, between-group effects were correlated with symptom severity scores within the CSA-related PTSD group. Post-hoc, between-group effects were correlated with symptom severity scores within the CSA-related PTSD group.

## Results

### Sample characteristics

The means and standard deviations of demographic and clinical characteristics are presented in Table 1. The majority of participants was female (85.7%). The adolescents with CSA-related PTSD had a significantly lower TIQ (mean 99.3, SD 8.8) than controls (mean 107.0, SD 8.3) [t(47)= −3.120, p = 0.003]. In addition, adolescents with PTSD (mean 16.4, SD 2.0) were significantly older than controls (mean 15.2, SD 1.6) [t (47)=−2.338, p = 0.024]. As expected, there was a significant difference between the total scores on the A-DES and TSCC between adolescents with CSA-related PTSD and healthy non-traumatised controls, with a higher mean total score for CSA-participants. There was one adolescent in the CSA-related PTSD group with comorbid anxiety disorder not otherwise specified. Among the adolescents with CSA, 2 adolescents were on SSRI treatment and 2 used amphetamines, the latter were not used on the day of the MRI scan.

**Table 1:** Sample characteristics: Means and SD of age, TIQ, total scores of ADES, total scores of TSCC, and numbers per gender and PDS ratings.

		CSA-related PTSD	SD	Controls	SD	*Group comparison*
		N = 21		N = 28		p
Gender (f: m)		18:3		24:4		
Age (years)		16.4	2.1	15.2	1.6	0.024
TIQ		99.3	8.8	107.0	8.3	0.003
PDS	Pre/mid pubertal	1		8		0.020
	Late pubertal	10		14		
	Post pubertal	10		6		
A-DES^a^	Total score	72.6	58.8	22.3	19.7	0.001
TSCC^a^	Total score	47.9	2.5	17.4	13.6	0.000 0,000002

Abbreviations: CSA, Childhood Sexual Abuse; PTSD, Post Traumatic Stress Disorder; TIQ, Total Intelligence Quotient; PDS, Puberty Development Scale; A-DES, Adolescent Dissociative Experiences Scale; TSCC, Trauma Symptom Checklist for Children.

^a^4 did not complete the A-DES and TSCC.

### Cortical thickness, surface area and volume

Differences in cortical thickness, surface area and volume between adolescents with CSA-related PTSD and non-traumatised controls were assessed in the selected ROI. Overall, no significant effect of group was found in the MANCOVAs for cortical thickness (F(10,35) = 1.130, p = 0.369) (Table 2), surface area (F(10,35) = 0.850, p = 0.586) (Table 3) or volume (F(10,35) = 1.050, p = 0.425) (Table 4). Excluding the 2 CSA-participants that were using medication from the analyses did not change the results.

**Table 2:** Contributions of each ROI’s cortical thickness.

ROI	Cortical thickness (mm)		F-value	P-value
	CSA-related PTSD	Controls		
***Left hemisphere***				
Caudal anterior cingulate cortex	3.13 (0.32)	3.10 (0.23)	0.872	0.355
Rostral anterior cingulate cortex	3.26 (0.20)	3.25 (0.21)	0.043	0.837
Middle temporal gyrus	3.03 (0.20)	3.12 (0.13)	2.495	0.121
Superior temporal gyrus	2.94 (0.22)	3.00 (0.15)	0.186	0.668
Medialorbitofrontal cortex	2.70 (0.23)	2.76 (0.13)	1.337	0.254
***Right hemisphere***				
Caudal anterior cingulate cortex	2.92 (0.32)	3.05 (0.27)	1.334	0.254
Rostral anterior cingulate cortex	3.21 (0.26)	3.28 (0.23)	0.473	0.495
Middle temporal gyrus	3.17 (0.21)	3.24 (0.12)	0.366	0.548
Superior temporal gyrus	2.98 (0.18)	3.06 (0.20)	1.405	0.242
Medialorbitofrontal cortex	2.79 (0.25)	2.83 (0.19)	0.387	0.537

Abbreviations: CSA, Childhood Sexual Abuse; PTSD, Post Traumatic Stress Disorder.

**Table 3:** Contributions of each ROI’s surface area.

ROI	Surface area (mm^2^)		F-value	P-value
	CSA-related PTSD	Controls		
***Left hemisphere***				
Caudal anterior cingulate cortex	586.05 (78.39)	697.79 (186.17)	6.041	0.018
Rostral anterior cingulate cortex	774.38 (124.50)	844.39 (135.67)	2.078	0.156
Middle temporal gyrus	2962.71 (431.81)	2972.75 (310.94)	0.408	0.526
Superior temporal gyrus	3667.33 (492.02)	4776.43 (438.840)	1.375	0.247
Medialorbitofrontal cortex	1872.00 (249.75)	1918.14 (241.51)	0.083	0.774
***Right hemisphere***				
Caudal anterior cingulate cortex	707.00 (199.30)	789.61 (207.27)	3.424	0.071
Rostral anterior cingulate cortex	634.71 (149.35)	672.39 (133.16)	0.735	0.396
Middle temporal gyrus	3301.52 (416.79)	3370.64 (410.00)	0.176	0.677
Superior temporal gyrus	3657.48 (453.63)	3706.11 (369.80)	0.316	0.577
Medialorbitofrontal cortex	1815.00 (179.90)	1851.89 (212.56)	0.230	0.634

Abbreviations: CSA, Childhood Sexual Abuse; PTSD, Post Traumatic Stress Disorder.

**Table 4:** Contributions of each ROI’s volume.

ROI	Volume (mm^3^)		F-value	P-value
	CSA-related PTSD	Controls		
***Left hemisphere***				
Caudal anterior cingulate cortex	1837.66 (329.66)	2153.61 (542.50)	3.548	0.066
Rostral anterior cingulate cortex	2518.97 (400.07)	2737.07 (443.40)	1.720	0.196
Middle temporal gyrus	8976.41 (1450.26)	9268.04 (1009.68)	0.994	0.324
Superior temporal gyrus	11329.50 (1454.24)	10792.39 (1716.97)	0.070	0.793
Medialorbitofrontal cortex	5029.51 (659.21)	5290.88 (701.20)	1.294	0.261
***Right hemisphere***				
Caudal anterior cingulate cortex	2055.94 (382.21)	2391.11 (575.40)	4.574	0.038
Rostral anterior cingulate cortex	2016.65 (391.04)	2196.90 (407.44)	1.879	0.177
Middle temporal gyrus	10430.92 (1342.12)	10950.74 (1531.57)	0.558	0.459
Superior temporal gyrus	10894.72 (1387.98)	11353.58 (1475.05)	0.611	0.439
Medialorbitofrontal cortex	5072.61 (688.89)	5248.09 (633.89)	1.548	0.220

Abbreviations: CSA, Childhood Sexual Abuse; PTSD, Post Traumatic Stress Disorder.

Also, excluding the one CSA-participant with sufficient PTSD symptoms, but limited interference from the analyses, did not change the results. As the CSA-related PTSD group had a significant lower TIQ than the controls, we report correlations with IQ within groups in the Supplementary Material Table S1. There were no significant correlations. There were significant correlations between PDS scores and age (Pearson’s r.632; p 0.001) and between PDS scores and ICV (Pearson’s r-0.298; p 0.005). There was no significant correlation between age and ICV (Pearson’s r-0.250). The variance inflation factor (VIF) between these three variables varied between 1.07 and 1.66, which indicates that there was low multicollinearity. As age, ICV and PDS were all included as covariates in the primary model, we did not perform any post hoc analysis in which one of these covariates was omitted. In addition, in post hoc analysis we tested for associations between structural brain measures and TSCC and ADES scores. These analyses only showed a significant overall correlation between regional volume and TSCC score. However, this result failed to reach significance when correcting for multiple comparisons. (Supplementary Material Table S2). Post hoc, we performed a power analysis based on comparable studies in the literature and concluded that the effect size is in the range of comparable studies. (Suplementary Material Analysis A1).

### Whole brain analysis

The exploratory whole-brain analysis did not reveal any overall differences in cortical thickness, surface area and volume between CSA-related PTSD and controls (cortical thickness (F(1,44) = 1.093, p = 0.656), surface area (F(1,44) = 0.885, p = 0.706) or volume (F(1,44) = 3.596 p = 0.339)). Also, there were no significant regional differences when corrected for multiple comparisons.

## Discussion

This study investigated cortical thickness, surface area and volume in a group of adolescents with CSA-related PTSD and a group of healthy non-traumatised controls. In contrast to our hypothesis, we found no significant difference between the two groups on any cortical morphometric measure in the ventromedial prefrontal cortex, anterior cingulate gyri, middle temporal gyri and superior temporal gyri.

To our knowledge this is the first study investigating cortical thickness in a group of minors with childhood sexual abuse. Up to this moment, cortical thickness studies in traumatised children and adolescents are scarce and mostly report smaller cortical thickness, but in a broad variety of regions. There are many methodological differences that hamper direct comparison of these studies, including selection of participants and controls, type of abuse, imaging parameters and methodology. There is one other study in minors, investigating traumatised adolescents with and without PTSD, which reported no abnormalities in cortical thickness in the insula, ACC, amygdala, CC and hippocampus^36^. However, in contrast to our study, this study included various types of trauma.

Our previous VBM study in the same population of the EPISCA project found smaller volumes of the dorsal ACC compared to healthy controls^22^. The current study does not implicate the ACC, but has used a different technique with a different delineation of cortical areas, which might explain the discrepancy in results.

Studies in traumatised adults, mostly in male war veterans with PTSD, consistently show reduced cortical thickness in several regions, such as the ACC and mPFC. In traumatised minors smaller ACC volume is not consistently reported^13^. Remarkably, however, the only study in a sample of adult women with sexual abuse related PTSD by Landré *et al*., similar to our study found normal cortical thickness compared to healthy controls^33^. The authors hypothesize that apart from gender, the specific type of trauma (i.e. sexual abuse versus combat related) might explain this difference with most other studies on trauma in adults. Interestingly, Heim *et al*. studied the cortical thickness in a sample of adult women with different forms of childhood abuse. They found in their adult sample that childhood sexual abuse was specifically associated with thinning of the somatosensory cortex representing the clitoris and the surrounding female genital area. In contrast to our study, they used a vertex based whole brain analysis and their sample consisted of adult women with CSA with and without depression or PTSD, potentially including resilient women^62^.

Apart from the methodological heterogeneity of studies, the dynamics of growth and development typically appearing in the brains of children and adolescents may in part explain the equivocal results in the literature. (See for example Weems *et al*. for the development of the amygdala in youth in relation to stress^63^).

In general, there is a growing awareness that different types of adversity may have different neurobiological sequelae and subsequent psychiatric and somatic disorders^64,65^. For instance, dysregulation of the immune system has been suggested by recent research as a possible biological mediator between adverse childhood experiences (ACE) and adulthood pathology. Baumeister *et al*., in a recent meta-analysis, conclude that there is strong evidence for the impact of ACE on the inflammatory immune system, where specific types of trauma (sexual, physical or emotional abuse) differentially impact on specific inflammatory markers and potentially pathogenic pro-inflammatory phenotypes associated with physical and mental illnesses^66^. The increased immune activation might be caused by changes in epigenetic regulation of gene expression. This appears plausible because of the considerable evidence for association of modifications of HPA- and neuroplasticity-related methylation patterns with childhood trauma^67^. Trauma specific research could thus contribute to more explicit implementation of interventions that reduce long term risks of childhood adversity and help defining specific treatments for children and adult survivors with psychopathology.

We believe that our study has several strengths. Although this was not an inclusion criterion, all participants in our study met DSM-IV criteria for PTSD, signalling that it is a clinically robust group. This study is part of the EPISCA project, in which we investigated the same groups with other structural and functional neuroimaging techniques that yielded results that were in line with the existing literature^19–24^. The homogeneous and well circumscribed sample of adolescents with CSA-related PTSD is a strength of this study. Some limitations should be mentioned. The CSA-related PTSD group in this study was significantly older than the control group and also more advanced in pubertal development. Longitudinal studies of typical adolescent brain development, however, show little change in most of our ROIs (mPFC, ACC, middle/sup temp gyrus) within the age range of participants of both groups^68^. Furthermore, we controlled for age and pubertal development in our analyses. In the statistical analysis we also controlled for total intracranial volume. Full-scale IQ measures were significantly lower in the CSA-related PTSD group than in the control group. As PTSD has been shown to supress IQ values, groups might originally have been more equal with respect to intellectual ability^69^. Brain development as well as the reaction to trauma is known to be susceptible to gender influences. We could not address this topic because our participants were mainly girls. Finally, as in our study several of the perpetrators of the CSA adolescents were family members, it was not possible to reliably assess timing and frequency of the traumatic experience retrospectively.

In conclusion, we found no differences in cortical measures between a group of adolescents with CSA-related PTSD and healthy non-traumatised controls. These findings support the suggestion that different types of adversity may have different neurobiological sequelae and subsequent psychiatric and somatic disorders. As childhood trauma is a highly relevant issue for society with severe consequences for psychological and general health, more and preferably longitudinal research into the neurobiological sequelae is needed.

## Supplementary information

Supplementary Material.
