# Supplementary material for: Preserved cortical thickness, surface area and volume in adolescents with PTSD after childhood sexual abuse
Source: Sci Rep. 2020 Feb 24;10:3266. doi: 10.1038/s41598-020-60256-3 (PMC7039962; doi:10.1038/s41598-020-60256-3)
Supplement: Supplementary file 1 — Supplementary Material. [file 41598_2020_60256_MOESM1_ESM.doc]

**Preserved cortical thickness, surface area and volume in adolescents with PTSD after childhood sexual abuse**

Mirjam A. Rinne-Albers, M.D.

Charlotte P. Boateng, M.D.

Steven J. van der Werff, M.Sc.

Francien Lamers-Winkelman, Ph.D.

Serge A. Rombouts, Ph.D.

Robert R. Vermeiren, M.D., Ph.D.

Nic J. van der Wee, M.D., Ph.D.

Supplementary Material Table S1. **MANCOVA: Correlation of IQ**

CSA Group: 21

Thickness (F(10,10) =1.917, p=0.160) (Wilks’ Lambda)

SA (F(10,10) = 1.755, p=0.194) (Wilks’ Lambda)

Volume (F(10,10) = 1.324, p=0.333) (Wilks’ Lambda)

| **Thickness** | **F-value** | **P-value** |
| --- | --- | --- |
|  |  |  |
| *Left hemisphere* |  |  |
| Caudal anterior cingulate cortex | ,049 | ,827 |
| Rostral anterior cingulate cortex | 1,182 | ,290 |
| Middle temporal gyrus | 2,824 | ,109 |
| Superior temporal gyrus | 5,717 | ,027 |
| Medialorbitofrontal cortex | ,267 | ,611 |
| *Right hemisphere* |  |  |
| Caudal anterior cingulate cortex | 1,247 | ,278 |
| Rostral anterior cingulate cortex | 2,009 | ,173 |
| Middle temporal gyrus | 12,388 | ,002 |
| Superior temporal gyrus | 2,620 | ,122 |
| Medialorbitofrontal cortex | ,689 | ,417 |

| **Surface area** | **F-value** | **P-value** |
| --- | --- | --- |
|  |  |  |
| *Left hemisphere* |  |  |
| Caudal anterior cingulate cortex | ,474 | ,499 |
| Rostral anterior cingulate cortex | ,044 | ,835 |
| Middle temporal gyrus | ,124 | ,729 |
| Superior temporal gyrus | ,036 | ,852 |
| Medialorbitofrontal cortex | ,067 | ,799 |
| *Right hemisphere* |  |  |
| Caudal anterior cingulate cortex | 1,632 | ,217 |
| Rostral anterior cingulate cortex | ,018 | ,896 |
| Middle temporal gyrus | ,383 | ,543 |
| Superior temporal gyrus | ,014 | ,907 |
| Medialorbitofrontal cortex | ,159 | ,694 |

| **Volume** | **F-value** | **P-value** |
| --- | --- | --- |
|  |  |  |
| *Left hemisphere* |  |  |
| Caudal anterior cingulate cortex | ,453 | ,509 |
| Rostral anterior cingulate cortex | ,475 | ,499 |
| Middle temporal gyrus | ,457 | ,507 |
| Superior temporal gyrus | 1,062 | ,316 |
| Medialorbitofrontal cortex | 1,400 | ,251 |
| *Right hemisphere* |  |  |
| Caudal anterior cingulate cortex | 3,597 | ,073 |
| Rostral anterior cingulate cortex | ,770 | ,391 |
| Middle temporal gyrus | ,043 | ,838 |
| Superior temporal gyrus | ,573 | ,458 |
| Medialorbitofrontal cortex | ,460 | ,506 |

Control group: 28

Thickness (F(10,17) =0.747, p=0.811) (Wilks’ Lambda)

SA (F(10,17) = 1.399, p=0.261) (Wilks’ Lambda)

Volume (F(10,17) = 0.998, p=0.482) (Wilks’ Lambda)

| **Thickness** | **F-value** | **P-value** |
| --- | --- | --- |
|  |  |  |
| *Left hemisphere* |  |  |
| Caudal anterior cingulate cortex | 1,653 | ,210 |
| Rostral anterior cingulate cortex | ,006 | ,940 |
| Middle temporal gyrus | ,180 | ,675 |
| Superior temporal gyrus | ,013 | ,911 |
| Medialorbitofrontal cortex | 2,138 | ,156 |
| *Right hemisphere* |  |  |
| Caudal anterior cingulate cortex | ,799 | ,380 |
| Rostral anterior cingulate cortex | 4,186 | ,051 |
| Middle temporal gyrus | ,030 | ,865 |
| Superior temporal gyrus | ,057 | ,814 |
| Medialorbitofrontal cortex | ,270 | ,608 |

| **Surface area** | **F-value** | **P-value** |
| --- | --- | --- |
|  |  |  |
| *Left hemisphere* |  |  |
| Caudal anterior cingulate cortex | 1,023 | ,321 |
| Rostral anterior cingulate cortex | 1,640 | ,212 |
| Middle temporal gyrus | 1,264 | ,271 |
| Superior temporal gyrus | 2,347 | ,138 |
| Medialorbitofrontal cortex | ,129 | ,723 |
| *Right hemisphere* |  |  |
| Caudal anterior cingulate cortex | ,008 | ,927 |
| Rostral anterior cingulate cortex | ,525 | ,475 |
| Middle temporal gyrus | ,624 | ,437 |
| Superior temporal gyrus | ,010 | ,922 |
| Medialorbitofrontal cortex | ,001 | ,970 |

| **Volume** | **F-value** | **P-value** |
| --- | --- | --- |
|  |  |  |
| *Left hemisphere* | ,403 | ,531 |
| Caudal anterior cingulate cortex | 1,921 | ,177 |
| Rostral anterior cingulate cortex | ,009 | ,926 |
| Middle temporal gyrus | ,861 | ,362 |
| Superior temporal gyrus | 1,856 | ,185 |
| Medialorbitofrontal cortex | ,055 | ,817 |
| *Right hemisphere* |  |  |
| Caudal anterior cingulate cortex | 2,293 | ,142 |
| Rostral anterior cingulate cortex | ,081 | ,779 |
| Middle temporal gyrus | ,510 | ,482 |
| Superior temporal gyrus | ,037 | ,850 |
| Medialorbitofrontal cortex | ,403 | ,531 |

Supplementary Material Table S2. **MANCOVA: Correlation with ADES/TSCC within CSA group**

ADES: subjects = 18

Thickness (F(10,7) =0.503, p=0.843) (Wilks’ Lambda)

SA (F(10,7) = 0.549, p=0.812) (Wilks’ Lambda)

Volume (F(10,7) = 0.878, p=0.588 (Wilks’ Lambda)

| **Thickness** | **F-value** | **P-value** |
| --- | --- | --- |
|  |  |  |
| *Left hemisphere* |  |  |
| Caudal anterior cingulate cortex | 4,565 | ,048 |
| Rostral anterior cingulate cortex | 4,418 | ,052 |
| Middle temporal gyrus | ,102 | ,754 |
| Superior temporal gyrus | ,175 | ,681 |
| Medialorbitofrontal cortex | 2,216 | ,156 |
| *Right hemisphere* |  |  |
| Caudal anterior cingulate cortex | ,015 | ,903 |
| Rostral anterior cingulate cortex | ,248 | ,625 |
| Middle temporal gyrus | ,133 | ,720 |
| Superior temporal gyrus | ,632 | ,438 |
| Medialorbitofrontal cortex | ,172 | ,684 |

| **Surface area** | **F-value** | **P-value** |
| --- | --- | --- |
|  |  |  |
| *Left hemisphere* |  |  |
| Caudal anterior cingulate cortex | ,028 | ,869 |
| Rostral anterior cingulate cortex | 1,559 | ,230 |
| Middle temporal gyrus | 7,195 | ,016 |
| Superior temporal gyrus | ,875 | ,363 |
| Medialorbitofrontal cortex | 3,560 | ,077 |
| *Right hemisphere* |  |  |
| Caudal anterior cingulate cortex | 2,959 | ,105 |
| Rostral anterior cingulate cortex | ,653 | ,431 |
| Middle temporal gyrus | 3,568 | ,077 |
| Superior temporal gyrus | 2,994 | ,103 |
| Medialorbitofrontal cortex | 2,478 | ,135 |

| **Volume** | **F-value** | **P-value** |
| --- | --- | --- |
|  |  |  |
| *Left hemisphere* |  |  |
| Caudal anterior cingulate cortex | ,783 | ,389 |
| Rostral anterior cingulate cortex | ,233 | ,636 |
| Middle temporal gyrus | ,815 | ,380 |
| Superior temporal gyrus | 4,315 | ,054 |
| Medialorbitofrontal cortex | ,384 | ,544 |
| *Right hemisphere* |  |  |
| Caudal anterior cingulate cortex | 2,146 | ,162 |
| Rostral anterior cingulate cortex | 1,156 | ,298 |
| Middle temporal gyrus | ,699 | ,415 |
| Superior temporal gyrus | 2,792 | ,114 |
| Medialorbitofrontal cortex | 1,548 | ,231 |

TSCC: subjects = 18

Thickness (F(10,7) = 0.758, p=0.666) (Wilks’ Lambda)

SA (F(10,7) = 2.069, p=0.173) (Wilks’ Lambda)

Volume (F(10,7) = 4.790, p=0.025) (Wilks’ Lambda) (multiple comparisons = 0.05/3)

| **Thickness** | **F-value** | **P-value** |
| --- | --- | --- |
|  |  |  |
| *Left hemisphere* |  |  |
| Caudal anterior cingulate cortex | 8,370 | ,011 |
| Rostral anterior cingulate cortex | 2,858 | ,110 |
| Middle temporal gyrus | ,250 | ,624 |
| Superior temporal gyrus | ,055 | ,817 |
| Medialorbitofrontal cortex | ,451 | ,511 |
| *Right hemisphere* |  |  |
| Caudal anterior cingulate cortex | ,109 | ,746 |
| Rostral anterior cingulate cortex | 1,014 | ,329 |
| Middle temporal gyrus | ,029 | ,867 |
| Superior temporal gyrus | ,003 | ,957 |
| Medialorbitofrontal cortex | ,180 | ,677 |

| **Surface area** | **F-value** | **P-value** |
| --- | --- | --- |
|  |  |  |
| *Left hemisphere* |  |  |
| Caudal anterior cingulate cortex | ,604 | ,448 |
| Rostral anterior cingulate cortex | 1,687 | ,212 |
| Middle temporal gyrus | 16,670 | ,001 |
| Superior temporal gyrus | 1,251 | ,280 |
| Medialorbitofrontal cortex | 2,154 | ,162 |
| *Right hemisphere* |  |  |
| Caudal anterior cingulate cortex | 5,167 | ,037 |
| Rostral anterior cingulate cortex | ,489 | ,494 |
| Middle temporal gyrus | 6,541 | ,021 |
| Superior temporal gyrus | 3,030 | ,101 |
| Medialorbitofrontal cortex | 4,118 | ,059 |

| **Volume** | **F-value** | **P-value** |
| --- | --- | --- |
|  |  |  |
| *Left hemisphere* |  |  |
| Caudal anterior cingulate cortex | ,568 | ,462 |
| Rostral anterior cingulate cortex | ,383 | ,545 |
| Middle temporal gyrus | 1,035 | ,324 |
| Superior temporal gyrus | 15,345 | ,001 |
| Medialorbitofrontal cortex | 1,194 | ,291 |
| *Right hemisphere* |  |  |
| Caudal anterior cingulate cortex | 2,539 | ,131 |
| Rostral anterior cingulate cortex | 1,359 | ,261 |
| Middle temporal gyrus | 3,134 | ,096 |
| Superior temporal gyrus | 7,222 | ,016 |
| Medialorbitofrontal cortex | 2,949 | ,105 |

Supplementary Material Analysis A1. **Poweranalysis**

N = 21 CSA, 28 controls (total = 49)

Sensitivity: compute required effect size: given alpha 0.05, power 0.95 and total sample size 49. Number of groups = 2, response variables = 10. Effect size = 0.63.

Meta-analysis by O’Doherty (1), medium to large effect sizes (0.5-0.8) (1). Number of groups =2, response variables = 1. Total sample size: 60.

(1) O'Doherty DC, Chitty KM, Saddiqui S, Bennett MR, Lagopoulos J. A systematic review and meta-analysis of magnetic resonance imaging measurement of structural volumes in posttraumatic stress disorder. Psychiatry Res. 232[1], 1-33. 4-30-2015.
